# Surveillance for Sri Lankan cassava mosaic virus (SLCMV) in Cambodia and Vietnam one year after its initial detection in a single plantation in 2015

**DOI:** 10.1371/journal.pone.0212780

**Published:** 2019-02-22

**Authors:** Nami Minato, Sophearith Sok, Songbi Chen, Erik Delaquis, Iv Phirun, Vi Xuan Le, Dharani D. Burra, Jonathan C. Newby, Kris A. G. Wyckhuys, Stef de Haan

**Affiliations:** 1 Agrobiodiversity Research Area, Asia Regional Office, International Center for Tropical Agriculture (CIAT), CGIAR Research Program on Roots Tubers and Bananas, Hanoi, Vietnam; 2 Tropical Crops Genetic Resources Institute (TCGRI), Chinese Academy of Tropical Agricultural Sciences (CATAS), Danzhou, Hainan, China; 3 Department of Industrial Crops, General Directorate of Agriculture (GDA), Phnom Penh, Cambodia; 4 Plant Protection Research Institute (PPRI), Vietnam Academy of Agricultural Sciences, Hanoi, Vietnam; 5 Decision and Policy Analysis Research Area, Asia Regional Office, International Center for Tropical Agriculture (CIAT), Hanoi, Vietnam; 6 International Joint Research Laboratory on Ecological Pest Management, Fuzhou, China; Shanghai Institutes for Biological Sciences, CHINA

## Abstract

Cassava mosaic disease, one of the ten most economically important crop viral diseases in the world, was first reported in Southeast Asia from a single plantation in Cambodia in 2015. To determine the presence and incidence of Sri Lankan cassava mosaic virus (SLCMV) one year after first detection, a total of 6,480 samples from 419 fields were systematically collected from cassava production areas across Cambodia (3,840 samples; 240 fields) and Vietnam (2,640samples; 179 fields) in the 2016 cropping season. Using PCR-based diagnostics, we identified 49 SLCMV-infected plants from nine fields, representing 2% of the total number of fields sampled. Infected fields were geographically restricted to two provinces of Eastern Cambodia, while no infection was detected from any of the other sampled sites in either country. Symptom expression patterns in infected plants suggested that SLCMV may have been transmitted both through infected planting materials, and by *Bemisia tabaci*, the known whitefly vector of SLCMV. In addition, 14% of virus infected plants did not express typical symptoms of cassava mosaic disease on their leaves, highlighting that molecular-based validation is needed to confirm the presence of SLCMV in the field. None of the owners of the SLCMV-infected fields indicated acquired planting materials from the plantation in Ratanakiri where SLCMV was first reported. The surveillance baseline data generated for both countries is discussed in light of future options to control and manage cassava mosaic disease.

## Introduction

In 2016 Sri Lankan cassava mosaic virus (SLCMV) was reported for the first time infecting cassava in Southeast Asia [1]. This report consisted of positive virus detection from a single commercial plantation with symptomatic plants in Ratanakiri province in Eastern Cambodia in May, 2015. Prior to this positive identification, Southeast Asia had been considered free of cassava mosaic disease (CMD). Given the negative effects on production and economic returns of CMD in other settings [2–6], an alert to notify the presence of the disease in the region was warranted [7]. At the time a window of opportunity for effective disease control through eradication or quarantine seemed apparent, as presence of the disease was assumed to be restricted to a limited geographic area.

Like other cassava mosaic geminiviruses (CMGs), such as African cassava mosaic virus (ACMV) and Indian cassava mosaic virus (ICMV), Sri Lankan cassava mosaic virus (family *Geminiviridae*, genus *Begomovirus*) is a causative agent of CMD. In Asia, the occurrence of CMGs has historically been restricted to South Asia, with the exception of a report of ICMV on *Jatropha curcas* in Singapore [8]. The recent report of SLCMV in Cambodia expands on previous identifications in Sri Lanka and India [1, 9–11]. Like other CMGs, SLCMV is transmitted by the whitefly *Bemisia tabaci* (Gennadius) (Hemiptera: Aleyrodidae), and through the movement of infected planting materials [12]. With ACMV, plants grown from infected cuttings are more seriously affected than those infected by the whitefly vector [13]. Although whitefly vectoring has contributed greatly to CMD outbreaks across Africa, an epidemiological study in India demonstrated that primary spread in that context occurred through the use of infected planting materials, with whitefly vectoring playing a secondary role [14]. The evidence for virus-induced quality degeneration of planting materials, associated yield decline, and consequent economic effects is abundant for CMD in Africa [2,15]. The level of yield decline experienced depends on several factors, including varietal responses, symptom severity, and means of propagation [13,16–18]. Experiences in Africa showed yield loss from CMD to be greater in cassava grown from infected cuttings (55–77%) than in plants infected later through whitefly vectoring (35–60%) [2].

Little is known about the effects of SLCMV on cassava productivity compared to its African counterpart. In India average losses of 30% from CMD have been reported [4]. In South Asia SLCMV is known to mutate quickly and to spread more aggressively than ICMV occupying a wider host range, especially in the Euphorbiaceae and Solanaceae plant families [9,19–22]. The frequency of CMG infection in host plants is generally low, resulting in only cultivated cassava being epidemiologically significant as a virus host in Africa [14], although additional research is needed to identify the primary host(s) of CMG in Asia.

Throughout mainland Southeast Asia, cassava (*Manihot esculenta* Crantz) is largely grown by millions of farmers as an industrial crop [23,24]. Over the past several decades, the crop’s cultivated area has rapidly expanded in Vietnam and Cambodia, with 569,900 and 684,070 ha cultivated in Vietnam and Cambodia, respectively, in 2016 [25,26]. The harvested area in Cambodia expanded more than 49 times over 15 years from 2001 to 2016 [25,27]. The rapid expansion of the crop with unchecked and wide-reaching movement of planting materials has been accompanied by the co-expansion of several biotic constraints, including mealybugs and cassava witches’ broom disease [28]. The increased demand for planting materials through informal channels without the introduction of phytosanitary controls, coupled with the propensity of *B*. *tabaci* to rapidly spread CMD causing viruses at local scale, puts the cassava sector at risk of a regional epidemic.

To determine the current distribution and incidence of SLCMV, we embarked on a baseline surveillance initiative in both Cambodia and Vietnam in the cropping season from 2016 to 2017. The surveillance initiative reported in this manuscript was accompanied by a parallel seed systems survey evaluating characteristics of cassava planting stem exchange behaviors, including networks of physical exchange throughout the study site [29]. Cassava is propagated commercially through the use of planting stems collected from mature plants at harvest, which are cut into 15-20cm sections at time of planting. Stems therefore take on the role of ‘seed’ in Southeast Asian cassava production. While the original introductory event of SLCMV into Southeast Asia remains unclear, systematic monitoring of the presence, incidence, and spread of the disease beyond its initial infection area is now extremely important in order to determine its severity, set a baseline for the disease’s early spread, and provide recommendations towards possible mitigation measures. Here we report on the first systematic bi-national SLCMV surveillance effort in Cambodia and Vietnam.

## Materials and methods

### Design of the bi-national survey

**Fig 1:**
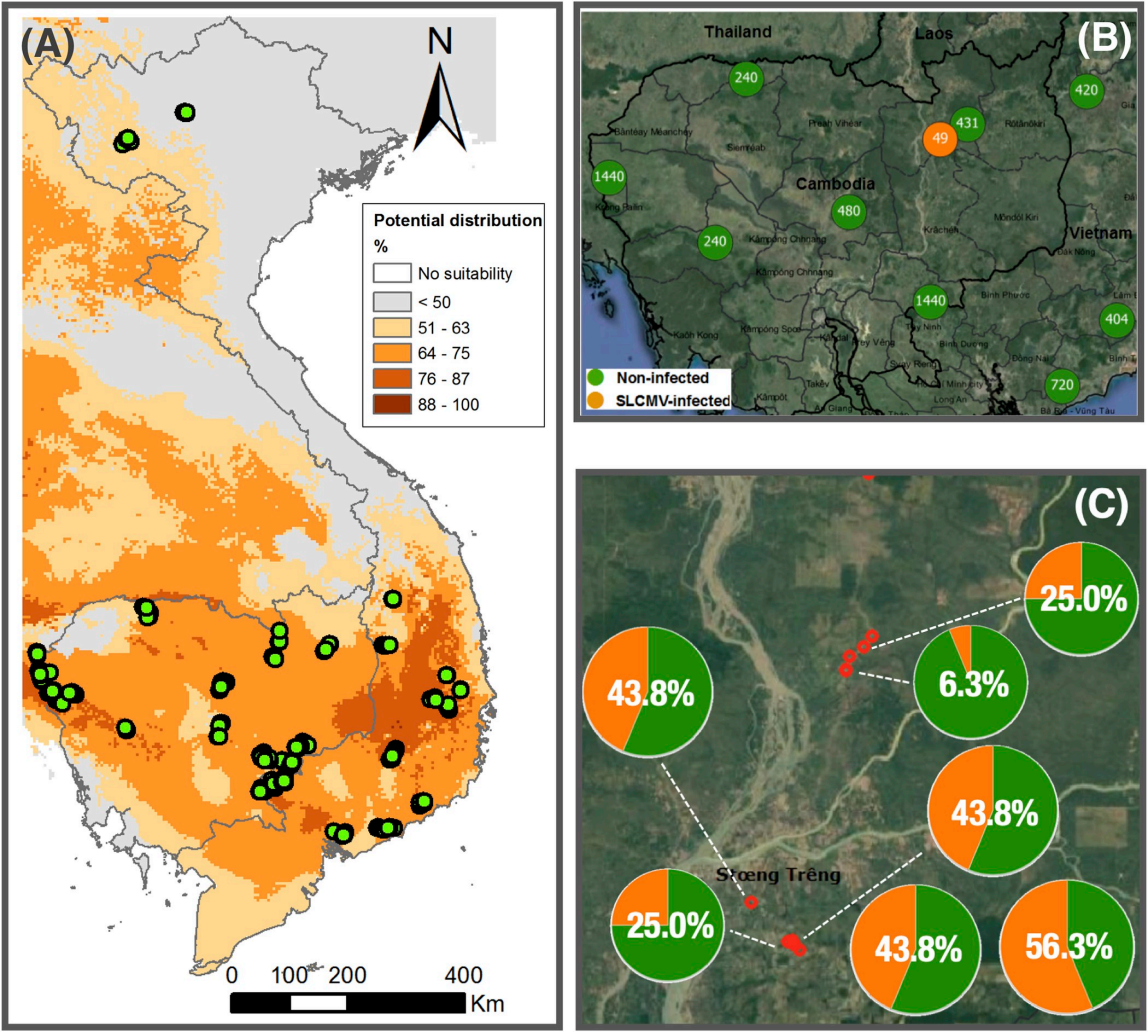
Study sites of the 2016 survey and geographical distribution of Sri Lankan cassava mosaic virus (SLCMV). (A) Location of sampling sites in Cambodia and Vietnam (green dots) with potential distribution of *Bemisia tabaci* (%) adapted from [32,33]. (B) Map of clustered sampling points for SLCMV detection in Cambodia and Southern Vietnam. Orange circles indicate sites of SLCMV infection, while green circles indicate sites with no detected infection. The number in each circle indicates the number of plants sampled for SLCMV diagnosis. (C) SLCMV within-field incidence in seven infected fields of Stung Treng province.

In order to investigate the geographical distribution of SLCMV we conducted a systematic nation-wide survey in Cambodia and Vietnam from November to December 2016. Districts with the largest cassava cropping areas were selected, following similar studies in other contexts [30,31]. For Vietnam, 15 districts were selected with the largest production areas of cassava (based on the 2014 census), and for Cambodia, 15 districts were selected with the highest production density (based on the latest available published estimate from FAO in 2011). An additional district was added in Eastern Cambodia, where SLCMV was first reported [1]. Where possible, this information was updated with production data from local authorities in the Northwest, Central Highlands, South Central Coast and Southwest regions of Vietnam, and across the Eastern and Western regions of Cambodia. Nation-wide sample collection was conducted in 8 provinces, 15 districts of Vietnam, and 11 provinces, 16 districts of Cambodia. In each district, 15 fields belonging to the same number of households were haphazardly chosen approximately equidistantly along the primary motorable road [30]. This resulted in a total of 419 fields and households, and 6,480 plants (**Table 1**). Matching global positioning system (GPS) coordinates were collected from every sampled field for geographical mapping. Study sites were located between 103.68082 and 108.91122 Eastern longitude and 10.72501 and 21.91563 Northern latitude in Vietnam, and between 102.34596 and 106.88632 Eastern longitude and 11.3989 and 14.2555 Northern latitude in Cambodia (**Fig 1(A)**).

**Table 1:** Sampling locations in Vietnam and Cambodia.

Country	ID	Province	District
**Vietnam**	**V1**	Son La	Thuan Chau
	**V2**	Yen Bai	Van Yen
	**V3**	Gia Lai	Chu Prong
	**V4**	Dak Lak	Mad Lak
	**V5**	Dak Lak	Eaker
	**V6**	Dak Nong	Dak Glong
	**V7**	Kon Tum	Sa Thay
	**V8**	Gia Lai	Krong Pa
	**V9**	Phu Yen	Song Hinh
	**V10**	Binh Thuan	Bac Binh
	**V11**	Binh Thuan	Ham Tan
	**V12**	Tay Ninh	Tan Bien
	**V13**	Tay Ninh	Tan Chau
	**V14**	Binh Thuan	Ham Thuan Nam
	**V15**	Dong Nai	Long Thanh
**Cambodia**	**K1**	Oddar Meancheay	Anlong Veaeng
	**K2**	Banteay Meanchey	Malai
	**K3**	Pailin	Sala Krau
	**K4**	Pailin	Pailin
	**K5**	Battambang	Kamrieng
	**K6**	Battambang	Phnum Proek
	**K7**	Battambang	Rattanak mondul
	**K8**	Pursat	Kravanh
	**K9**	Ratanakiri	Koun Mom
	**K10**	Steung Treng	Steung Treng
	**K11**	Kratie	Snoul
	**K12**	Tbong Khmun	Dambae
	**K13**	Tbong Khmun	Memot
	**K14**	Svay rieng	Romeas Haek
	**K15**	Kampong Thom	Sandan
	**K16**	Kampong Thom	Baray

### Geographical distribution maps

Survey locations in Cambodia and Vietnam were mapped with the potential distribution of whitefly *B*. *tabaci* [32,33], open access dataset. The geographical distribution of SLCMV-infected plants and clustered map were developed using QGIS.

### Sample collection

Young leaf tissue was sampled for virus diagnosis during the bi-national survey, from November to December 2016 during the local dry season [28]. For sampling of cassava leaves, two transects per field were selected, following an X-shape from border-to-border to cover the whole field, irrespective of its size [30]. Four plants were selected at approximately equidistant intervals in each half transect from the center of the field. About 100 mg of young leaf tissue was collected from the top of the canopy, from each of 16 individual plants per field, labelled with a unique identifying code, and placed in silica gel. When cassava plants had multiple primary stems, leaf tissue was collected from all of them. Leaf sampling was accompanied by photography, both of the whole plant and of its apex, using mobile tablet devices for visual symptom inspection. A database with coded images of each sampled field and plant was derived.

### DNA extraction

DNA was extracted from dried leaf tissue using the modified CTAB method [34]. Leaf tissue (~20mg dry weight) was ground with beads using a homogenizer. We then added 600 μL of CTAB buffer (30mM EDTA pH8.0, 0.1M Tris-HCl pH8.0, 1.2M NaCl, 3% CTAB and 1% β-mercaptoethanol), and incubated the solution for 60 minutes in a 65°C water bath. 400 μL of 24:1 chloroform: isoamylalcohol (CI) was added and incubated for 10 minutes after inversion. The supernatant was collected after centrifuging at 10,000 rpm for 10 minutes. An equivalent volume of CI was added again and the previous step repeated. A double volume of cold 100% ethanol was added to the solution and kept at -20°C for two hours. After centrifuging, the precipitate was rinsed with 70% ethanol and dissolved in distilled water. The extract was stored at 4°C for subsequent use in PCR.

### PCR assay & electrophoresis

SLCMV diagnosis was carried out using specific primers based on the *AC1* (*replicase*) gene of SLCMV reported by Duraisamy *et al*. [12]. Thermal cycling consisted of an initial denaturation at 94°C for 5 minutes, followed by 40 cycles of 2 minutes at 94°C, annealing at 55°C for 30 seconds, 72°C for 1 minute, followed by a final extension of 5 minutes at 72°C. PCR components were based on a total reaction volume of 25 μL, consisting of 1.25U of EasyTaq DNA Polymerase (AP111, Beijing TransGen Biotech, China), 0.2mM of dNTPs (Beijing TransGen Biotech), 0.2 μM of primers and 10x EasyTaq buffer (Beijing TransGen Biotech). Amplified PCR products were subjected to electrophoresis using a 1.0% agarose gel (Regular Agarose G-10, BIOWEST, France) stained with Gel Stain (GS101-01, Beijing TransGen Biotech) in 1x Tris-acetate/EDTA buffer (diluted from 50x TAE: T1060-500, Beijing Solarbio Science & Technology, China) and photographed under UV light.

### Sequencing and phylogenetic analysis

PCR products for subsequent sequencing were amplified using ExTaq (RR001; TaKaRa, Japan). Amplicons were directly sequenced and obtained DNA sequences were aligned using ClustalW implemented in MEGA7: Molecular Evolutionary Genetics Analysis version 7.0 (http://www.megasoftware.net/) [35] with available DNA-A sequences of SLCMV and ICMV from GenBank (http://www.ncbi.nlm.nih.gov), together with sequences obtained from the nine SLCMV isolates from this study (GenBank Accession Numbers: MH351658-MH351666). GenBank Accession Number: KT861468.1 is the equivalent sequence published by the first disease report of SLCMV in Cambodia. The phylogenetic tree was constructed using the maximum-likelihood method with 1,000 bootstrap replicates in MEGA7 on the basis of 1,004nt of *AC1* gene partial sequences, and the equivalent to each sequence from tomato mosaic virus (ToMV; GenBank ID: NC001938) was used as an out-group to root the tree.

### Seed systems survey

A parallel seed system survey (in this context ‘seed’ referring to cassava planting stems) was conducted with each of the households participating in the virus surveillance. Detailed methods of this accompanying study have been described by Delaquis *et al*. [29]. Herein we refer only to seed system survey results for those households with SLCMV infected plants in order to gain insight into potential sources of infection via planting material.

### Research ethics

Ethical review and approval of the interview method, data collection, and data handling protocols were obtained from the CIAT Institutional Review Board, and met CIAT—related guidelines for research involving human subjects.

## Results

### Presence and incidence of SLCMV in Cambodia and Vietnam

To determine the presence and the incidence of SLCMV, a total of 6,480 samples systematically collected from 419 fields across Cambodia and Vietnam in the 2016 cropping season were tested using PCR-based diagnostics. We found 49 SLCMV-infected plants in nine distinct fields, representing 2% of the total number of fields sampled. Positive samples were restricted to two provinces of Eastern Cambodia, while no infection was detected from any of the other sampled sites in either country (**Fig 1(A) and 1(B)**).

In Ratanakiri province of Cambodia, a total of two fields were found to be infected with SLCMV. Both were located in Koun Mom district, and had within-field infection rates of 37.5 and 25.0% (**Table 2**). The infected fields were located approximately 15 km away from the 2015 focal point of initial disease detection. SLCMV was also detected in Stung Treng, the neighboring province located to the West of Ratanakiri. The within-field incidence in Stung Treng ranged from 6.3 to 56.3% (**Table 2; Fig 1(C)**). Some of the infected fields were adjacent to each other, while in other cases non-infected fields separated infected ones from each other, suggesting that the distribution of infected fields in each province was not solely dependent on distance from the 2015 infection site. The overall field level incidence of SLCMV was 13.3 and 46.6% in Ratanakiri and Stung Treng provinces, respectively (**Table 2**). The most distant infected site relative to the location of first detection [1,7] was approximately 70 km away.

**Table 2:** Number of Sri Lankan cassava mosaic virus (SLCMV)-infected plants and infection rate in each field of Ratanakiri and Stung Treng provinces.

Field code		Number of SLCMV infected plants / total	Infection rate (%)
Koun Mom district, Ratanakiri province			
	K9F1	0/16	0
	K9F2	6/16	37.5
	K9F3	4/16	25.0
	K9F4	0/16	0
	K9F5	0/16	0
	K9F6	0/16	0
	K9F7	0/16	0
	K9F8	0/16	0
	K9F9	0/16	0
	K9F10	0/16	0
	K9F11	0/16	0
	K9F12	0/16	0
	K9F13	0/16	0
	K9F14	0/16	0
	K9F15	0/16	0
Stueng Traeng district, Stung Treng province			
	K10F1	4/16	25.0
	K10F2	7/16	43.8
	K10F3	0/16	0
	K10F4	0/16	0
	K10F5	1/16	6.3
	K10F6	4/16	25.0
	K10F7	0/16	0
	K10F8	0/16	0
	K10F9	0/16	0
	K10F10	0/16	0
	K10F11	0/16	0
	K10F12	0/16	0
	K10F13	7/16	43.8
	K10F14	7/16	43.8
	K10F15	9/16	56.3

### Symptom observations

**Fig 2:**
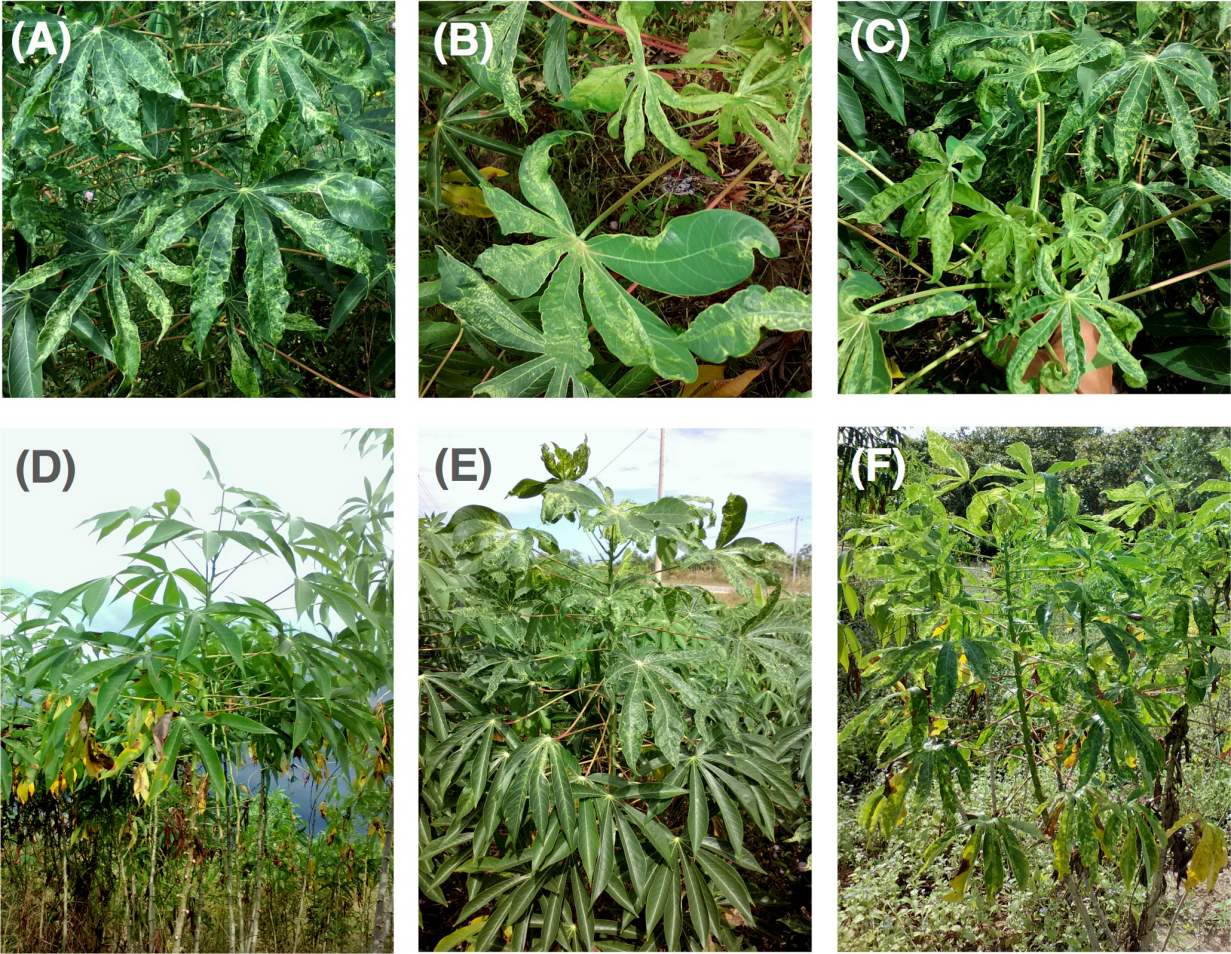
Symptoms observed on SLCMV positive plants identified in Cambodia. (A)-(C) Typical CMD symptoms on leaves, (A) mosaic, (B) deformation, and (C) curl. (D) Asymptomatic plant testing positive by PCR for SLCMV infection. (E) Plant with mosaic symptoms only on upper leaves and (F) plant with systemic mosaic symptoms.

Infected plants represented 0.8% of the total plants sampled, and corresponded to nine fields. The 49 cassava plants with confirmed SLCMV infection were reviewed in the photographic database to retrospectively check for visual disease symptoms. Of the infected plants, 83.7% exhibited at least one of the typical foliar symptoms of CMD, such as mosaic pattern, curl, and deformation (**Fig 2(A), 2(B) and 2(C)**). Eight of the nine infected fields contained plants with clearly distinguishable symptoms, although the most severe symptoms of stunted growth were not observed on those plants (**Table 3**). In contrast, 14.3% of infected plants did not display any visual symptoms of mosaic, curl or deformation. SLCMV-infected cassava plants exhibiting no typical CMD symptoms were detected in five of the nine infected fields (**Fig 2(D)**).

**Table 3:** Symptom development in SLCMV-infected plants.

Characteristics	All infected plants	Infected plants less than 6 MAP
	(N = 49)	(N = 34) *^1^
**Symptoms—no. (%)**		
** Whole plant**		
** **Stunting	0	0
** **Dieback	3 (6.1%)	1 (2.9%)
** Leaf on top of canopy**		
** **Mosaic	36 (73.5%)	27 (79.4%)
** **Curl	23 (46.9%)	16 (47.0%)
** **Deformation	39 (79.6%)	28 (82.4%)
** **Dieback	15 (30.6%)	11 (32.4%)
** **Blighting	23 (46.9%)	14 (41.2%)
**Pattern of symptom expression—no. (%)**		
** **Asymptomatic*^2^	7 (14.3%)	4 (11.8%)
** **Systemic*^3^	-	23 (67.6%)
** **Non-systemic*^4^	-	7 (20.6%)

*1: Infected plants that were planted after May 2016.

*2: Plants whose leaves did not show any of typical CMD symptoms, namely mosaic, curl, and deformation.

*3: Plants that showed CMD symptoms on lowest, older leaves.

*4: Plants that showed CMD symptoms only on the young, upper leaves.

Visual assessment of symptoms provided a reliable indication of virus infection in most cases of CMD documented in Africa [30], with infection via planting material causing mosaic symptoms visible on the lowest, older leaves, and insect vector transmission inducing mosaic symptoms only on younger, upper leaves emerging post virus transmission. Over 25% of the total number of plants diagnosed as infected by PCR did not exhibit visual leaf mosaic symptoms. Of the 34 SLCMV-infected plants sampled less than 6 months after planting, 67.6% exhibited symptoms on the lowest, oldest leaves (consistent with systemic infection via planting stems), while 20.6% exhibited mosaic symptoms only on young upper leaves (consistent with whitefly-vectored, non-systemic infection) (**Fig 2(E) and 2(F); Table 3**). Both Ratanakiri and Stung Treng province had fields containing both systemic and non-systemic plants. Leaf blight symptoms were observed on around 46% of the SLCMV-infected plants with moderate severity (**Table 3**), indicating co-infection of SLCMV and another bacterium causing cassava bacterial blight.

### Phylogeny

**Fig 3:**
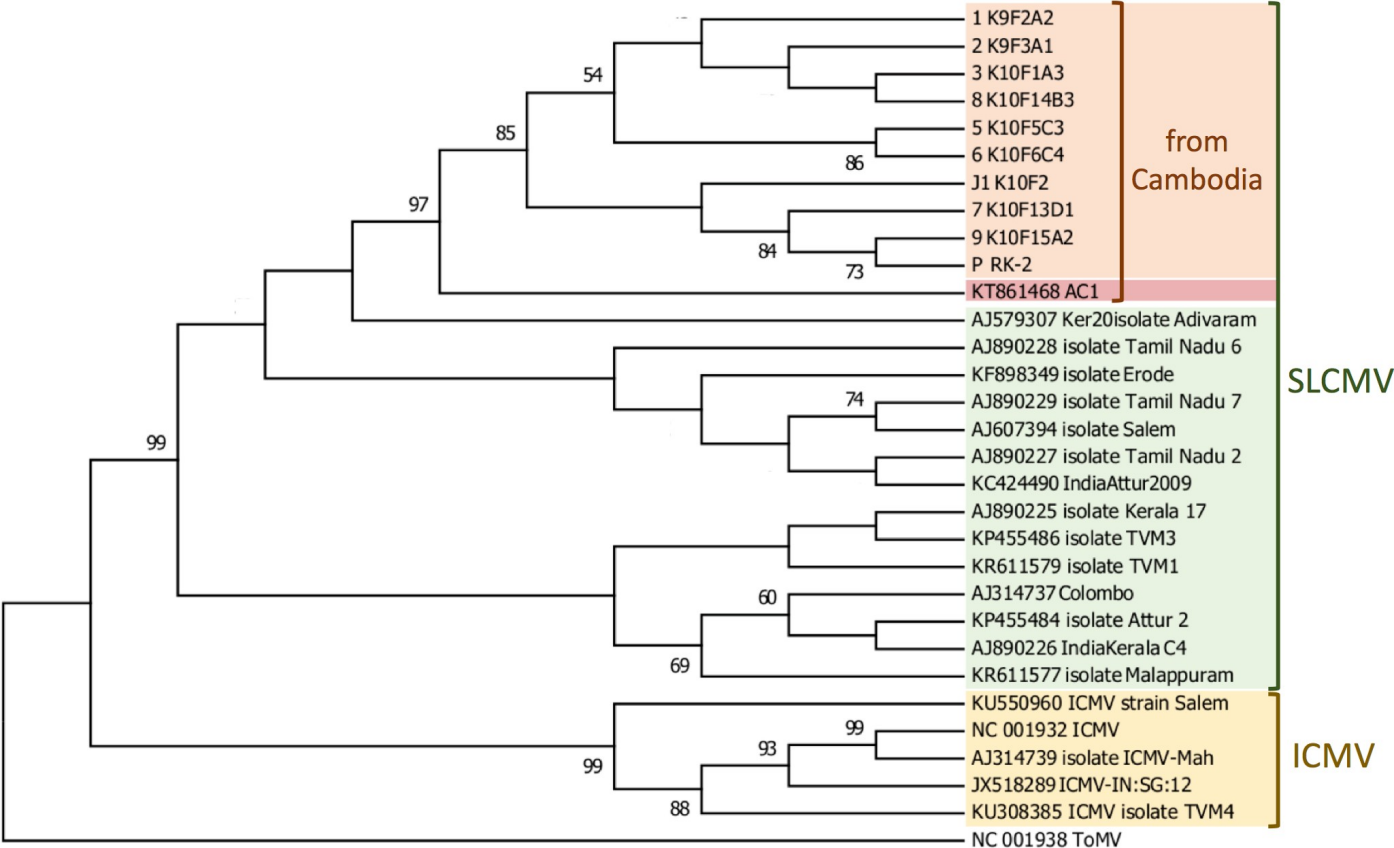
Maximum likelihood phylogenetic trees of the *AC1* gene (replication associated protein coding region) of DNA-A, including that of available Sri Lankan cassava mosaic virus and Indian cassava mosaic virus isolates. The equivalent to each sequence from tomato mosaic virus (ToMV) was used as an out-group to root the tree. The sequences were aligned, and phylogenetically re-constructed by MEGA7 software with 1,000 boot strap replications, obtained by the neighbor-joining method. Orange background indicates sequences of SLCMV isolates from this study: sequences 1 and 2 are from Ratanakiri province, and 3,5,6,7,8,9, J1, and P are from Stung Treng province. Red background indicates the sequence published by the first report of SLCMV in Cambodia.

Genetic relationships between SLCMV isolates were derived based on virus samples collected in all nine fields over two Cambodian provinces through maximum-likelihood phylogenetic analysis (**Fig 3; S1 Fig**). Sequences of the virus isolates were obtained from one virus infected plant per infected field. The resulting topology clearly indicates that SLCMV and ICMV fall into two distinct clusters. In comparison, between SLCMV isolates from this study and from previous studies in South Asia and Cambodia, all SLCMV isolates from our study were grouped separately from the first reported isolate in 2016 [1].

### Stem provenance in SLCMV-infected fields

The owners of each sampled field were interviewed to collect additional data on seed system characteristics [29]. Except for one household, all owners of the SLCMV-infected fields used farm-saved stems from the previous season (**Table 4**). One owner of infected fields from Stung Treng province procured planting materials from Kampong Cham, the adjacent province located to the Southwest of the infected site through a trader. No respondents acquired planting materials from the plantation in Ratanakiri, where SLCMV was first reported. Without exception surveyed SLCMV-infected fields were not greater than 2ha in area. Among the nine households owning the virus-infected fields, three of the respondents did not indicate that they faced general pest and disease problems on their farms, even though two of them had plants with clear CMD symptoms.

**Table 4:** Origin of planting materials in fields testing positive for SLCMV.

	Field Code	Infection rate (%)*^1^	Source of stems	Stems from*^2^	Surveyed field size (ha)	Month planted	Pest/Disease recognition *^3^
**Koun Mom district, Ratanakiri province**							
	K9F2	37.5	own stock from 2015	The same village	2	May 2016	Yes
	K9F3	25.0	own stock from 2015	The same field	1	May 2016	No
**Stueng Traeng district, Stung Treng province**							
	K10F1	25.0	own stock from 2015	The same village	0.1	May 2016	Yes
	K10F2	43.8	own stock from 2015 and trader	The same village and Kampong Cham province	0.4	Sep 2015	Yes
	K10F5	6.3	own stock from 2015	The same field	1	Oct 2015	No
	K10F6	25.0	own stock from 2015	The same field	0.5	Jul 2016	No
	K10F13	43.8	own stock from 2015	The same village	0.5	Apr 2016	Yes
	K10F14	43.8	Friend / neighbour / relative within the community	The same village	0.5	Mar 2016	Yes
	K10F15	56.3	own stock from 2015	The same village	0.1	Jun 2016	Yes

*1: Infection rate (%) of SLCMV was determined by PCR-based analysis.

*2: The origin of the stakes that were grown in farmers’ field during 2016 cropping season.

*3: Farmer’s recognition of general pests and/or diseases problems in their own field.

## Discussion

Our study provides the first systematic baseline assessment of SLCMV presence and incidence for both Cambodia and Vietnam after the virus was first positively detected in Cambodia. We show that SLCMV is not only present in the province where it was first detected, but also in the neighboring province of Stung Treng. In fact, levels of incidence observed in Stung Treng were much higher than Ratanakiri in terms of number of infected fields and within-field incidence rates (**Fig 1**; **Table 2**). While the exact mechanism and first location of introduction of SLCMV to Southeast Asia remains unclear, positive detections in our study remained confined to Eastern Cambodia. The range of distribution of the virus was up to 70 km away from the 2015 initial detection site reported by Wang *et al*. [1]. However, this doesn’t necessarily imply that the disease has spread from this point, because the original study only sampled a single location based on symptomatic observations, and did not attempt to ascertain the geographic extent of the infection. Our findings indicate that at the time of the study, SLCMV was still geographically restricted to a relatively confined region of Cambodia, thus improving the potential for preventative measures to limit its further spread. Such measures could include quarantine, eradication, or restrictions on plant movement.

Importantly, in 2016 we didn’t detect SLCMV in Central, West, South, or Northern Cambodia, or in Vietnam. However, there is a risk of rapid spread. Control measures and continued monitoring are essential going forward. Infected plants with both systemic and non-systemic symptoms were detected in both provinces, suggesting that the SLCMV infections observed in our study may have originated from both planting of infected stem cuttings, and by whitefly transmission. A better understanding of the interactions between SLCMV and common Southeast Asian cassava varieties is required, as the symptomatic distinction between systemic and non-systemic infections is mostly based on African experiences with the closely related ACMV group [30]. Although little is known about SLCMV’s specific epidemiology, we argue that lessons can be drawn from CMGs in both Africa and South Asia. The rate of transmission and geographical spread in these cases was dependent on diverse interacting factors, including whitefly populations, climatic conditions, and planting material movement patterns [2,36–38].

In Southeast Asia, the whitefly vector *B*. *tabaci* has a widespread complex with diverse species, and its potential distribution area covers nearly all of Cambodia and Southern Vietnam [32,39]. Virus spread data in Africa indicated that whitefly populations are able to carry CMGs over distances of up to 38 km in a year [40]. The presence of SLCMV in multiple fields in Eastern Cambodia offering adequate conditions for further spread to the surrounding area. Vector presence, suitable climatic conditions for vector reproduction, in combination with large areas of cassava monoculture, can potentially trigger a Southeast Asian pandemic.

In addition to whitefly vectoring, stem procurement networks will likely contribute to the further spread of SLCMV from Ratanakiri and Stung Treng to more distant locations. Regional trade networks of planting materials include long-distance connections covering up to 300 km [29], hence it is highly likely that SLCMV will also spread through stem movement, in addition to primary local infection through whiteflies. Stem procurement through specialized traders, because of its potential for long-distance planting material exchange, will likely contribute to the disease establishing itself more quickly in areas that are currently considered disease free [29]. In fact, during the season following our baseline study suspected SLCMV symptoms were observed in Tay Ninh province of Southern Vietnam [41], several hundred kilometers away from the 2016 infection sites reported in our study. Particular preventative attention should be paid to the movement of planting materials out of infected areas in order to keep the range of SLCMV restricted and to avoid a situation of multiple, geographically distant infection hotspots. We found that most of the infected fields in Eastern Cambodia were planted with farm-saved stakes from the previous season or sourced from within the village. This suggests an opportunity to contain the disease within its current restricted range distribution if adequate preventative strategies are adopted.

We found asymptomatic infection of SLCMV on 16% of positively diagnosed samples from multiple fields. This is particularly worrying because it suggests that the disease can spread undetected by visual inspection. In all documented cases in Africa, CMD induced chlorotic mosaic symptoms on the leaves of affected plants [40]. The general symptoms of CMD in South Asia are the same as those observed in Africa, but indeed masking of symptoms has also been reported in India [4,14]. The use of positively selected planting materials from symptomless plants had been widely recommended as a practical control measures for farmers to adopt [2,30,42]. Positive and negative selection based on symptom recognition are potentially one of the few readily applicable recommendations for smallholder farmers and extension agents alike, especially in the Southeast Asian context where resistant varieties are non-existent, and mobile diagnostic tools are currently inaccessible. Asymptomatic infection will complicate the monitoring of infected plants by visual inspection and, consequently, the development and introduction of simplified pocket diagnostic methods is urgently needed, just as in the case of human diseases [43]. Potential technologies exist [44,45], but their practical field-level application remains to be validated.

Short, medium, and long-term management strategies should be implemented to reduce further spread of SLCMV. Short-term measures should be attempted through quarantine, restrictions on plant movement, sanitation, and eradication of infected fields. Yet, the temporal window for such actions is limited, and benefits may be greatly reduced once serious infections are apparent in multiple hotspots and multiple sources of re-infection become common via planting materials or whitefly transmission [14]. In our survey, some of the owners of infected fields were not necessarily aware that the symptoms indicated the presence of a disease as opposed to varietal differences or weather-induced damages, despite the exhibition of clear CMD symptoms (**Table 4**). Since SLCMV is new in Southeast Asia, awareness raising and capacity building will be essential for disease management. Medium-term strategies should focus on seed sector development, particularly supply chains that are able to deliver disease free planting materials. Such efforts can potentially build on the strengths of the current seed systems (i.e., decentralized distribution) while trying to simultaneously address key shortcomings such as the lack of quality assurance [29]. The most promising long-term strategy for SLCMV management involves resistance breeding. Breeding programs in India, where ICMV and SLCMV are endemic, have made rapid progress in resistance breeding and the release of SLCMV resistant varieties [14,46]. Yet, in Southeast Asia there is currently little investment in cassava disease resistance breeding. Introducing known sources of resistance, screening of new breeding populations under intentional exposure, and eventually releasing SLCMV resistant varieties will require multi-year investments and consistent cooperation between regional bodies.

## Conclusions

We report on a baseline-level systematic bi-national survey of SLCMV presence and incidence in Cambodia and Vietnam one year after the disease was first reported in Eastern Cambodia. Since Southeast Asia contributes over 95% of global cassava exports [23], the potential negative impacts of SLCMV on cassava-based production systems are a major regional and global concern. The potential impacts of SLCMV threaten the precarious livelihoods of millions of smallholder farmers across Southeast Asia. At the time of our bi-national surveillance exercise, the 2016 range distribution of SLCMV remained restricted to Eastern Cambodia. Our finding of highest SLCMV levels in Stung Treng province, even when compared to the neighboring province of Ratanakiri, where the disease was first detected in 2015, suggest that the range of the outbreak is already beyond the province of initial detection.

Regional efforts for continuous monitoring and surveillance involving multiple stakeholders are required to systematically track disease spread internationally, update baseline knowledge, share information openly, and guide containment strategies. A centrally coordinated data platform with up-to-date information could provide such intelligence to inform preventative and control measures. Given the 2016 restricted range distribution we recommend that quarantine measures, restrictions on stem movement and eradication might still offer a means to control the disease. However, the window is likely very short, and decisive collective action is required.

Symptomatic observations suggest that cassava was likely infected through both whitefly and stem transmission. Whitefly potential distribution models [32] and recent research on cassava seed networks [29] clearly suggest that disease spread via both insect vectors and stem exchange pose a regional concern. This is likely aggravated by the fact that 16% of diseased plants were asymptomatic, highlighting the need for affordable field-level diagnostic tools beyond visual symptom recognition as a means to effectively monitor disease spread. In addition, in-depth surveys of whitefly prevalence and biotypes will be essential to modeling and handling local outbreaks. The arrival of SLCMV in Southeast Asia requires an integrated approach, in which diagnostic technologies, adapted advisory, regular monitoring, combined control strategies, and cross-sectoral coordination are essential components. Future surveillance can make use of the present baseline to benchmark the possible expansion of the disease.

## Supporting information

S1 Fig Maximum likelihood phylogenetic trees of the *AC1* gene. The equivalent to each sequence from tomato mosaic virus (ToMV) was used as an out-group to root the tree. The sequences were aligned, and phylogenetically re-constructed by MEGA7 software with 1,000 boot strap replications, obtained by the neighbor-joining method. Sequences 1 and 2 are from Ratanakiri province, and 3,5,6,7,8,9, J1, and P are from Steung Treng province.
(TIF)
