# Supplementary figures and images for: Surveillance for Sri Lankan cassava mosaic virus (SLCMV) in Cambodia and Vietnam one year after its initial detection in a single plantation in 2015
Source: PLoS One. 2019 Feb 22;14(2):e0212780. doi: 10.1371/journal.pone.0212780 (PMC6386488; doi:10.1371/journal.pone.0212780)

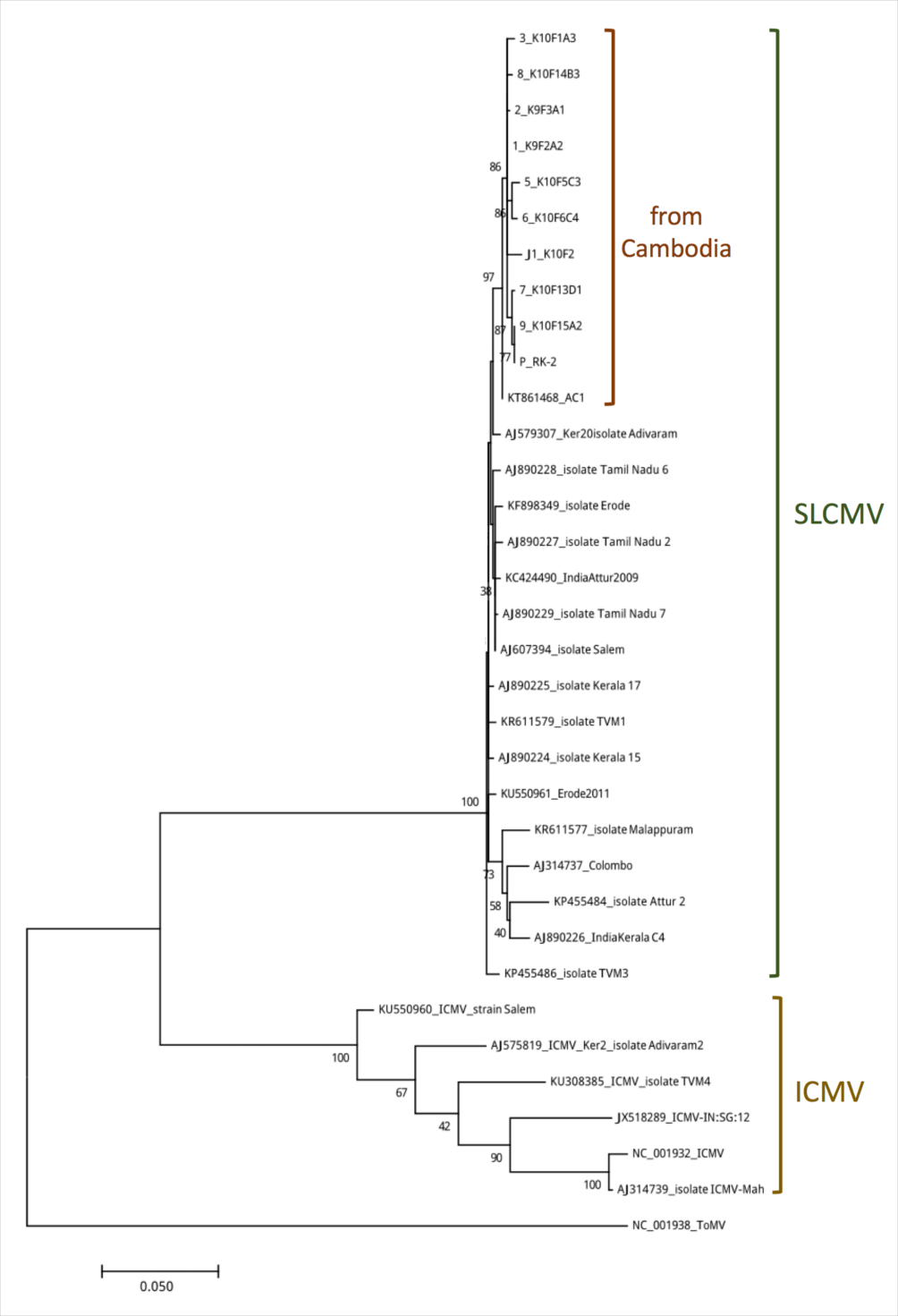

Supplement: S1 Fig — The equivalent to each sequence from tomato mosaic virus (ToMV) was used as an out-group to root the tree. The sequences were aligned, and phylogenetically re-constructed by MEGA7 software with 1,000 boot strap replications, obtained by the neighbor-joining method. Sequences 1 and 2 are from Ratanakiri province, and 3,5,6,7,8,9, J1, and P are from Steung Treng province. (TIF) [file pone.0212780.s001.tif]
